# Effect of Washing Times on the Quality Characteristics and Protein Oxidation of Silver Carp Surimi

**DOI:** 10.3390/foods11162397

**Published:** 2022-08-10

**Authors:** Xiaodi Zhang, Yiqi Zhang, Haochen Ding, Wenhai Zhang, Zhiyuan Dai

**Affiliations:** 1 Collaborative Innovation Center of Seafood Deep Processing, Zhejiang Province Joint Key Laboratory of Aquatic Products Processing, Institute of Seafood, Zhejiang Gongshang University, Hangzhou 310035, China; 2 Anjing Foods Group Co., Ltd., Xiamen 361022, China

**Keywords:** washing times, silver carp surimi, quality characteristics, protein oxidation

## Abstract

The aim of this work is to evaluate the effects of different washing times (zero (W0), one (W1), two (W2), and three (W3) times) on the physicochemical characteristics, gel property, and protein oxidation of silver carp surimi during 4 °C refrigeration. The results showed that the yield, types of fatty acids, redness (a*), total volatile basic nitrogen, and thiobarbituric acid reactive substances of the surimi tended to decrease, and the whiteness, pH, gel strength, and water retention tended to increase with the increase of washing times. Meanwhile, washing removed some fatty acids and the fatty acid species showed a decreasing trend. The FTIR spectra showed that washing did not change the functional group composition but changed the content of each group of the functional groups, while decreasing the proportion of *β*-sheet structures. Compared with the unwashed surimi, washing caused some of the immobilized water in the minced fish to be transferred to free water, and the water fluidity was enhanced. The washing enhanced the water holding capacity in the surimi gels, and the microstructure of the surimi gels was denser and delayed the protein oxidation during refrigeration. However, the difference between W2 and W3 surimi was not significant (*p* > 0.05). In practice, W2 can be used to produce surimi to improve its yield and reduce water consumption.

## 1. Introduction

Surimi has the advantages of high protein, low fat, and delicate taste, which is popular among consumers [1]. At present, the raw materials for the production of surimi are mainly seawater fish, but due to human overfishing and changes in the natural environment, marine economic fish resources cannot meet the human consumption demand for surimi products [2]. In recent years, the production of freshwater fish in China has increased significantly, and the development of freshwater fish for the production of surimi has become important research in the field of surimi processing.

Silver carp (*Hypophthalmichthys molitrix*), one of the four major fish species in China, is the main low-value freshwater fish in China, with a production of about 3.81 million tons in 2020 [3]. Silver carp is the best freshwater fish for producing surimi, with its short growth cycle, low price, and tender meat. The excess fat and protein in fish react and reduce the nutritional and organoleptic properties of the product [4]. In addition, protein oxidation can induce cross-linking reactions to denature proteins and reduce their functionality [5]. Therefore, washing is an essential process in the production of surimi, which removes impurities, fat, and water-soluble proteins [6] and thus improves the color, texture, and odor of surimi products [7]. The amount of washing times depends on the type of fish and washing solution. Granata, Flick Jr, and Martin [8] showed that producing 1 kg of surimi requires nearly 15 kg of water. A higher amount of washing solution can produce surimi with better quality, but it increases the waste-water discharge of the factory, decreases surimi yield, and also has an impact on the environment [6]. Therefore, moderate washing of surimi can maximize surimi yield and reduce water consumption.

In order to achieve sustainable and efficient development of the surimi processing industry, it is necessary to understand the effect of washing times on surimi [9]. However, there are few studies that are related to the effect of washing times on the quality of silver carp surimi. In view of this, we investigated the effect of washing times on the quality of surimi and evaluated the effect of different washing times on the physicochemical characteristics, gel property, and protein oxidation of silver carp surimi to provide a theoretical reference for the production of high quality surimi by loss reduction treatment.

## 2. Materials and Methods

### 2.1. Materials

Silver carp surimi was provided by Anjoyfood Co., Ltd. (Liaoning, China). A 10× phosphate buffered solution (PBS) and color Mixed Protein Marker (11–245 KDa) were purchased from Beijing Solarbio Science & Technology Co., Ltd. (Beijing, China). The 0.1 mol/L HCl standard titration solution, 10× electrode buffer, and bicarbonate reagent were purchased from Fuzhou Feixing Biotechnology Co. (Fuzhou, China). All other reagents were analytical grade and were purchased from Sinopharm Chemical Reagent Co., Ltd. (Shanghai, China).

### 2.2. Preparation of Surimi

The silver carp was divided into four groups for washing after harvesting, the first group of minced fish was not washed (W0), the second group was washed one time (W1), the third group was washed two times (W2), and the fourth group was washed three times (W3). The fish that were washed with five times their weight in tap water for 15 min, and the washing water was agitated and kept below 4 °C during the washing process. After washing, the surimi was centrifuged and plate shaped. The above operations were carried out in the surimi production workshop of Liaoning Anjing Food Co., LTD (Anshan, China). The four groups of surimi were placed at 4 °C, and each index was measured.

### 2.3. Proximate Analysis

In brief, the moisture content was measured using the oven-drying method. The protein content was estimated from nitrogen (N × 6.25) using constant Kjeldahl method. The fat content of the sample was extracted from the sample by Soxhlet using ether as solvent. The ash content of the sample was determined using the method Muffle furnace ashing [10]. All the values that were analyzed were expressed as a percentage on a wet weight basis.

### 2.4. Fatty Acid Composition

The fat of surimi was extracted and methylated according to the method of Li et al. [4]. The fatty acid composition of surimi was determined by gas chromatography coupled with a flame ionization detector (FID) (7890B, Agilent, Palo Alto, CA, USA). The injection port and detector temperatures were 260 °C. A HP-88 column (100 m × 0.25 mm) was used for determination. The column temperature was set at 180 °C for the first 20 min, and then increased to 200 °C at a rate of 0.5 °C/min. N_2_ was used as the carrier gas (flow rate: 1.5 mL/min). The air flow and H_2_ flow were 400 and 30 mL/min, respectively.

### 2.5. Determination of Whiteness

The whiteness was determined with a HunterLab XE Chroma Meter (Hunter Engineering Company, Bridgeton, MO, USA) according to the method of Xiong et al. [11], by measuring the L* (lightness), a* (redness), and b* (yellowness) values. A total of nine readings were made from the surface of the samples. The *whiteness* was calculated by the following:

*Whiteness* = 100 − [(100 − L*^2^) + a*^2^ + b*^2^]^1/2^ (1)

### 2.6. Determination of pH

The surimi was weighed for 5.0 g, then 45 mL of distilled water was added, and the sample was homogenized for 30 s at 8000 r/min. The pH was measured using a hand-held pH meter (Seven 2Go-S2, Mettler Toledo, Greenville, SC, USA) [12].

### 2.7. Chemical Analysis of Surimi

The total volatile base nitrogen (TVB-N) was estimated by the micro diffusion method. The 2-thiobarbituric acid-reactive substances (TBARS) of surimi was estimated spectrophotometric method. In brief, the surimi samples (5.0 g) were treated according to the method of Priyadarshini et al. [13] and reacted with 0.02 mol/L 2-thiobarbituric acid (TBA) by heating in a boiling water bath for 40 min. The absorbance of the supernatant was measured at 532 nm.

### 2.8. Preparation of Surimi Gels

The moisture content of the surimi samples was measured using a halogen moisture meter (SN-DHS-20A, Shampo Instruments Co., Shanghai, China), and the moisture content of the surimi was calculated and adjusted. The moisture of the surimi was adjusted to 80% and 2.5% sodium chloride was added. The surimi was placed in a mixer with a paddle attachment for 3 min. The surimi paste was stuffed into polyvinylidine casing (21 mm in diameter) and both ends of the casing were sealed tightly. Subsequently, the surimi pastes were incubated at 40 °C for 30 min and then heated at 90 °C for 20 min [14]. The gels were cooled in iced water and stored overnight at 4 °C prior to analysis. The gel casings were peeled prior to the test.

### 2.9. Determination of Gel Strength

The gel strength was determined according to the method that was described by Zhang et al. [15]. The gels were cut into cylinders (2.5 cm in height) prior to the test. A spherical probe (5 mm in diameter) was used to penetrate 15 mm into the samples at a speed of 1 mm/s by a gel strength analyzer (SD-700, Sun Scientific Co., Ltd., Tokyo, Japan). The breaking force (maximum breaking force, g) and deformation (cm) were determined on at least nine specimens per treatment. The gel strength (g·cm) is the product of breaking force and deformation.

### 2.10. Determination of Water Holding Capacity (WHC)

The surimi gels (5.0 g) were weighed into centrifugation tubes. The WHC was performed by the sample as described by Jin et al. [16]. The WHC was expressed as percentage of water that was retained with respect to the water that was present in the gel prior to centrifugation.

### 2.11. Determination of Low Field Nuclear Magnetic Resonance (LF-NMR)

The LF-NMR of samples was measured according to the method of Yin et al. [17] using an NMR analyzer (MesoMR23-060H-I, Niumag analytical instrument Co., Suzhou, China). Approximately 5.0 g of cylindrical surimi gel was placed into an NMR tube (size: 2 cm in diameter × 4 cm in height). The Carr Purcell Meiboom Gill (CPMG) pulse sequence was used to show the transverse relaxation time (*T*_2_).

### 2.12. Fourier Transform Infrared (FTIR) Spectroscopy

We performed FTIR according to the method of Zhang, Dai, Zhang, Dong, and Hu [18]. The surimi gels were freeze-dried and mixed with potassium bromide (KBr). The mixture was ground uniformly and then compressed into a tablet. FTIR spectroscopy was carried out in a spectrometer (IN10, Thermo Fisher Scientific, Waltham, MA, USA).

### 2.13. Scanning Electron Microscopy (SEM)

We performed microstructure observation according to the method of Zhou et al. [1] and Shi et al. [19]. The freeze-dried surimi gel samples were sublimated and gilded. The microstructures were observed by SEM (Sigma 300, Zeiss, Oberkochen, Germany).

### 2.14. Preparation of Myofibrillar Protein (MP)

MP was prepared from according to the method of Liu, Chen, Kong, Han, and He [20].

### 2.15. Determination of Carbonyl Content

The carbonyl group reacts with 2,4-dinitrophenylhydrazine to form the red 2,4-dinitrophenylhydrazone, with a characteristic absorption peak at 370 nm. The carbonyl content was analyzed by an assay kit (Nanjing Jiancheng Technology Co. Ltd., Nanjing, China).

### 2.16. Determination of Total Sulphydryl Group Content

Total sulphydryl group content was determined according to the method of Balange, and Benjakul [21] with slight modifications. To 0.5 mL sample solution (5.0 mg/mL), 4.5 mL of buffer A (pH 6.8, containing 10 mmol/L EDTA, 8 mol/L urea, 1% SDS, and 0.2 mol/L Tris-HCI buffer) were added. To 4 mL of the mixture, 0.5 mL of buffer B (pH 8.0, containing 0.1% DTNB and 0.2 mol/L Tris-HCI buffer) was added and incubated at 40 °C for 30 min. A blank was conducted by replacing the sample with 0.6 mol/L NaCl. The absorbance was measured at 412 nm (Spectra MAX 190, Molecular Devices Co., Sunnyvale, TX, USA) and sulfhydryl group content was calculated using the extinction coefficient of 13,600 mol/L^−1^cm^−1^.

### 2.17. SDS-Polyacrylamide Gel Electrophoresis (SDS-PAGE)

Protein patterns of the surimi were analyzed by SDS-PAGE according to the method of Zhang et al. [22]. In brief, the surimi samples (3.0 g) were mixed with 5% SDS solution (30 mL), homogenized, and held at 85 °C for 1 h. After centrifugation (3500 r/min, 15 min) the supernatant was added to an equal volume of 1× loading buffer, dispersed and then boiled in a water bath for 3 min. Protein patterns of surimi samples (15 μL) were loaded onto polyacrylamide gels comprised of a 10% running gel and a 4% stacking gel and subjected to electrophoresis using an electrophoresis apparatus (BIO-RAD Co., Hercules, CA, USA). The gels were stained with 0.125% Coomassie brilliant blue R-250 and decolorized in a solution containing 65% acetic acid and 25% ethanol until the bands were clear.

### 2.18. Statistical Analysis

The colors and gel strengths of the samples were run in 9 replicates, and the rest of experiments were run in triplicate. The data are presented as the means ± standard deviations of duplicate measurements and subjected to analysis of variance (ANOVA) using SPSS 22.0 Statistics (SPSS Inc., Chicago, IL, USA). The means were compared by the Duncan’s multiple range test at the 95% significant level (*p* < 0.05).

## 3. Results and Discussion

### 3.1. Basic Composition of Surimi

The basic composition of silver carp surimi is shown in Table 1. The results showed that with the increase of washing times, the moisture content of the surimi increased from 73.14% to 76.30%. Washing caused the hydration of myofibrillar protein of surimi, and the moisture content of the surimi increased. The moisture content of the surimi after washing was significantly higher than that of the unwashed surimi (*p* < 0.05). After repeated washing, the protein content of the surimi decreased. The protein content of the surimi in W2 (22.87%) and W3 (22.21%) was significantly lower than that in W0 (24.71%) and W1 (24.19%). This result is consistent with a previous study that was conducted by Li et al. [4], in which it was found that in the washing process, a large number of water-soluble proteins (myosinogen, hemoglobin, myoglobin, and so on) are removed, as well as part of the volatile amino compounds. The fat content of the surimi decreased significantly from 1.52% (W0) to 1.10% (W3) in the washing process, but there was no significant difference between the fat content of W2 and W3 (*p* > 0.05). Silver carp belong to low-fat fish, the overall fat content is low, but part of the fat is removed in the washing process. The fats in surimi are oxidized and interact with proteins to deteriorate the quality of the surimi [16]. After washing, most of water-soluble protein and part of the fat could be removed from surimi, but there was no significant difference in the ash content (*p* > 0.05). Producing high-quality surimi requires high protein, high myofibrillar protein, low fat, and adequate moisture [16]. The appropriate number of washing times retains some of the sarcoplasmic proteins and endogenous transglutaminases in the surimi, which can improve the gel strength of surimi gels.

### 3.2. Fatty Acid Composition of Surimi

As can be seen from Table 2, the types of fatty acids in the surimi decreased with the increase of washing times, and the washing removed some saturated fatty acids. The fatty acid species of surimi decreased from 20 to 15 after three washing times, and the washing reduced the fatty acid species of surimi. Due to the low density of fat, in the upper layer of the washing water, the fatty acid species decreased gradually as the washing times increased fat removal. A similar result was reported by Li et al. [4], who likewise found that washing reduced the types of fatty acids in surimi. Surimi contains a large amount of C18:1 monounsaturated fatty acids, the most abundant polyunsaturated fatty acids are C20:4, followed by EPA and DHA, which are very important fatty acids for humans. With the increase of washing times, the change of EPA and DHA content is small, and the surimi with different washing times have s high nutritional value.

### 3.3. Physicochemical Characteristics of Surimi

The influence of different washing times on the physicochemical characteristics of surimi is shown in Table 3. The yield of surimi decreased with the increase of washing times, from 88.58% to 76.73%. Among them, the yield of twice-rinsing surimi was increased by 5.92% compared with three-rinsing surimi. In the traditional surimi production process, three times of cold-water washing is usually carried out to remove impurities (including sarcoplasmic protein, blood, pigment, and fat) in fish mince, thereby improving the whiteness and quality of the surimi. The redness (a*) and whiteness of unwashed surimi (W0) were 1.44 and 65.23, respectively. The a* and whiteness of W3 were −1.08 and 71.96, respectively. With the increase of washing times, the a* of surimi showed a decreasing trend, while the whiteness showed an increasing trend. Myoglobin is a heme protein that is soluble in water and has an important effect on the formation of fish minced color and rancidity [7]. During the washing process of surimi, most of the myosinogen, myoglobin, hemoglobin, and other compounds are removed, thereby reducing the a* of surimi and improving the whiteness of surimi [23]. Although increasing the washing times improved the whiteness of the surimi, there was no significant difference between the W2 and W3 groups (*p* > 0.05). At the same time, washing can cause a waste of water and a loss of nutrients in the surimi, so it is important to choose the appropriate number of surimi washing times in production.

The pH of the surimi increased from 6.32 (W0) to 6.83 (W3) with an increasing number of washing times. Water-soluble acidic compounds (such as free acidic amino acids and lactic acid) are removed from the surimi [24], so the pH of surimi increased significantly (*p* < 0.05). Jin et al. [16] noted that the pH has a profound effect on the physical characteristics of meat, such as moisture content, tenderness, and color. An increase in the pH may lead to a decrease in the fat content of surimi. When the pH is not at the isoelectric point of myofibrillar protein, neutral and polar fats in minced meat can be removed and fat in minced meat can be released [25]. However, during the washing process, both alkaline and acidic substances in the surimi can be washed away, so that the surimi reaches a new pH balance after washing [23].

TVB-N is mainly composed of trimethylamine, dimethylamine, and ammonia. Protein oxidation that is caused by bacterial metabolism in fish leads to an increase in TVB-N, which is associated with fish freshness [26]. Generally speaking, the acceptable limit of TVB-N for consumers is 25–35 mg N/100 g, higher than the limit indicates that the minced meat has deteriorated [27]. The TVB-N of W0 was 6.57 mg N/100 g, indicating that the quality of fresh fish was better. With the increase of washing times, the TVB-N of surimi gradually decreased (*p* < 0.05). Therefore, the washing times of surimi can be appropriately increased in industrial production to improve the quality of surimi [28].

Fat oxidation can reduce the color, flavor, texture, and nutritional value of surimi. W0 contained more fat and endogenous pro-oxidants and, therefore, had the highest TBARS (0.183 mg/kg) [7]. With the increase of washing times, the TBARS of surimi decreased significantly (*p* < 0.05). In general, the oxidation of fat and myoglobin in minced meat occurs simultaneously and synergistically [29]. W3 had the lowest TBARS in this study (0.086 mg/kg, *p* < 0.05), which might be related to the removal of some fat and oxidized components in fish mince during washing.

Gel strength is an important index to evaluate the gel quality of surimi. As shown in Table 2, the gel strength of W0 was the lowest (330.20 g·cm, *p* < 0.05), which may be because the heat-induced proteases and fats interfered with the cross-linking of myosin in the gel matrix of surimi [6]. With the increase of washing times, some substances that interfere with myosin cross-linking were removed, so the gel strength of surimi significantly improved (*p* < 0.05). Kong, Geng, Gao, and Li [30] observed that washing could improve the gel properties of surimi, but too many washing times would also reduce the gel properties of surimi, which should be controlled at two to three times. In this study, it was found that gel strength of surimi of W2 and W3 (>400 g·cm) was higher than the Chinese standard of AAA surimi (≥400 g·cm), which indicated that the surimi washing two times still met the standard.

The WHC of surimi gel is related to myofibrillar protein molecular structure and charge change [24]. When the pH of minced meat is close to the isoelectric point of myofibrillar protein (pH = 5.5), the protein agglutinates, leading to the release of water molecules and thus affecting the formation of surimi gel [23]. With the increase of washing times, the WHC of the surimi gel increased significantly (*p* < 0.05, 90.90–92.87%) compared to W0 (88.46%). After washing, the pH of the surimi deviated from the isoelectric point of myofibrillar protein, thus improving the WHC of surimi gel, which was consistent with the results of Baxter and Skonberg [31]. Karthikeyan Dileep, and Shamasundar, [32] showed that compared with unwashed *Nemipterus Japonicus* surimi, the WHC of the washed three times surimi gel was 10% higher than that of the control. The difference in WHC depends on the stability of the protein network, and components (such as fat) that interfere with the stability of the protein network may be removed in washing [31].

In summary, washing treatment could improve the physicochemical characteristics of surimi, but there was no significant difference between W2 and W3 in color, TVB-N, TBARS, gel strength, and water holding capacity (*p* < 0.05). It can be estimated that by reducing the number of surimi rinsing steps from three times to two times, the discharge of rinsing wastewater can be reduced by at least 30%, which can effectively reduce the cost of surimi wastewater treatment and improve the utilization rate of fish resources.

### 3.4. FTIR Spectra

**Figure 1:**
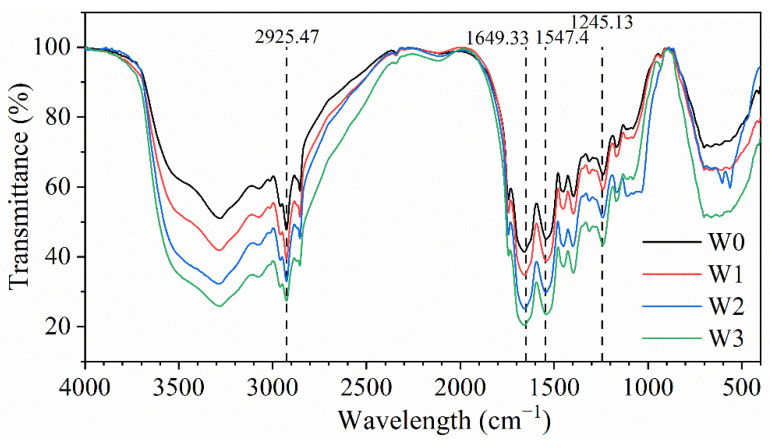
The FTIR spectra of surimi gel with different washing times.

The FTIR spectra of surimi gel with different washing times are shown in Figure 1. The peak at 2980–2850 cm^−1^ represented the asymmetric stretching of CH_2_ with a characteristic absorption peak of 2925.47 cm^−1^, usually occurring in the aliphatic side chain of proteins. The peak at 1700–1600 cm^−1^ was the amide I band, representing the stretching vibration of C=O. And the peak at 1350–1200 cm^−1^ was associated with vibration of C-O and C-O-O functional groups [33]. The absorption spectra of the four groups of surimi in the figure were similar, indicating that the washing times had no effect on the composition of the functional groups in the surimi gels [7]. However, the intensity of the absorption peaks of the surimi samples increased with the increase in washing times, which indicated that the washing process affected the content of the functional groups in the surimi gels.

The most characteristic spectral band in myofibrillar protein was the amide I band, which reflected hydrogen bonding patterns, dipole interactions, and the geometry of the protein polypeptide backbone [33]. In this study, we compared the Gaussian fitting curves of the amide I band of surimi gels with different washing times (Figure S1) to obtain the secondary structure content of surimi gel proteins, and the results are shown in Table 4. The *α*-helix, *β*-sheet, *β*-turn, and random coil structure contents of the unwashed surimi gels (W0) were 30.87%, 44.06%, 12.18%, and 12.88%, respectively. Washing increased the *α*-helix content and significantly decreased the *β*-sheet content (*p* < 0.05) of surimi gels, indicating that the surimi gels were more compact after washing [13]. Liu et al. [34] also found that compared to unwashed minced, the *β*-sheet content of washed surimi gel decreased by 13% and the *β*-turn content increased by 39%, while the changes in *α*-helical and random coil content were not significantly different (*p* > 0.05). A similar result was reported by Yuan et al. [35], who likewise found that *β*-sheet content decreased with the increase of washing times.

### 3.5. Low Field NMR Relaxation

**Figure 2:**
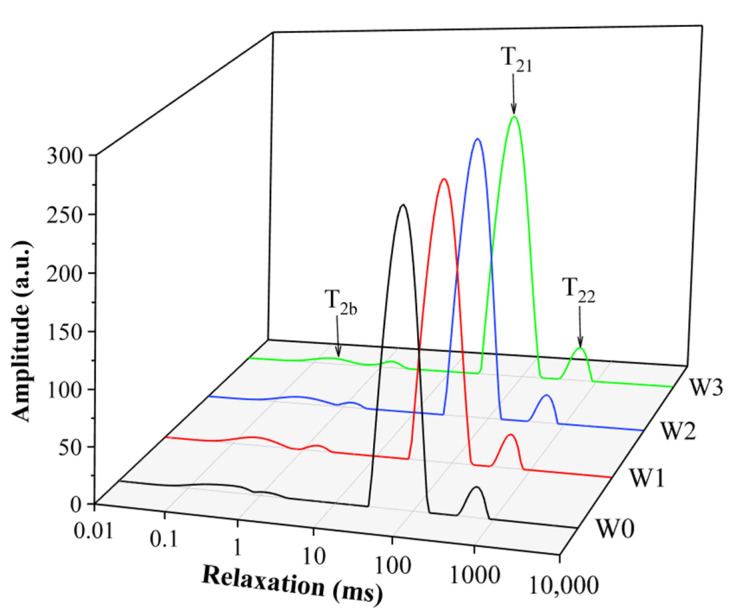
*T*_2_ relaxation times of surimi gel with different washing times.

LF-NMR analysis results could characterize changes in water mobility and distribution in surimi gels, which correlated with protein denaturation and aggregation [36]. The *T*_2_ relaxation time distribution results of surimi gel with different washing times are shown in Figure 2 and Table 5. As relaxation components, *T*_2b_ represented the protein binding water that was tightly bound with macromolecules (bound water), *T*_21_ represented the water embedded in the protein network structure (immobilized water), and *T*_22_ represented the free water outside the spatial structure of surimi gel (free water) [37]. The results showed that there was more immobilized water and less bound water and free water in the surimi gel, and most of the water in the surimi gel was bound within the spatial structure, which was consistent with the findings of Wang et al. [38] and Pan et al. [39]. Compared to the unwashed mince, washing increased the percentage of *T*_2b_ and *T*_22_ and decreased the percentage of *T*_21_ in the surimi gel, indicating that the interaction between water and protein molecules was weaker, some immobilized water was transferred to free water, and the mobility of water was enhanced [38].

### 3.6. Microstructures of Surimi Gel

**Figure 3:**
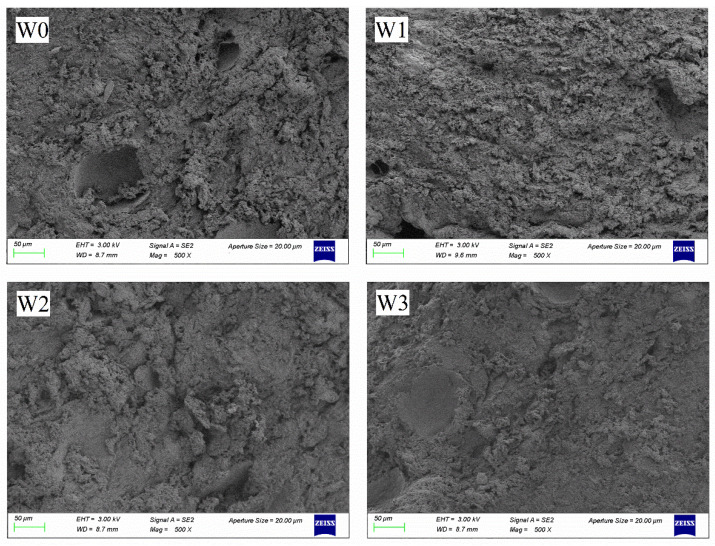
Microstructures (500× magnifications) of surimi gel with different washing times.

The microstructure of surimi gels with different washing times are shown in Figure 3. All the surimi gels had a reticular structure and the cross-linking of myofibrillar protein gave the surimi gels a spatial structure that conferred some elasticity [7]. Bertram, Meyer, Wu, Zhou, and Andersen [40] demonstrated that the water in the protein structure of the surimi gels was locked and created pores. The W0 had a loose and rough surface with varying pore sizes, which was also the reason why the W0 had the lowest gel strength and WHC [21]. Compared with W0, the pores of surimi gels gradually became smaller with increasing washing, indicating that washing improved the spatial structure of surimi gels to some extent. Since washing removed substances that interfered with the gel structure (such as fat), it allowed myofibrillar protein to aggregate more efficiently, thereby inducing protein cross-linking and forming a denser gel network [21]. The W2 and W3 gels were denser and had similar microstructures. The smaller pores in W2 and W3 were uniformly distributed in the surimi gels, which had a finer and more uniform spatial structure that bound more water, giving the W2 and W3 groups of surimi a greater water retention capacity, which was consistent with their higher WHC, gel strength, water content, and peak area of *T*_22_ [39]. Kang et al. [41] showed that differences in the gel microstructure might be related to the pH of the gel and that interactions such as binding and aggregation of proteins in the gel network were dependent on the temperature and pH.

### 3.7. Protein Oxidation of Surimi during 4 °C Refrigeration

**Figure 4:**
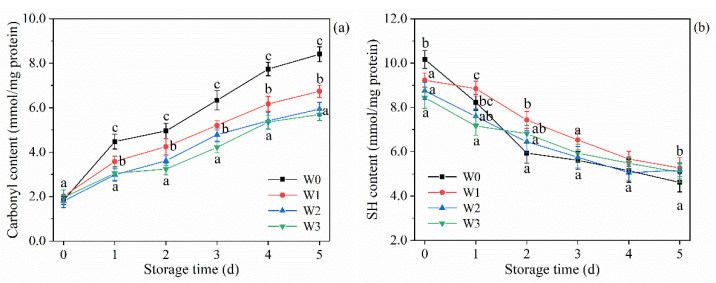
Changes of surimi with different washing times on the carbonyl group (**a**) and SH content (**b**) during 4 °C refrigeration. Different letters denote the significant difference (*p* < 0.05).

The extent of protein oxidation could be characterized by the carbonyl content. During storage, carbonyl compounds were produced following the breakage of amino acid side chains and peptide chains, or they could be produced by the covalent binding of proteins to oxidation products of sugars or fats in foods, making the carbonyl content an important indicator of the level of muscle protein oxidation [35]. Changes of the surimi with different washing times on the carbonyl group of the surimi during 4 °C refrigeration are shown in Figure 4a. At 0 d, there was no significant difference in the carbonyl content of the four groups of surimi (1.79–2.10 mmol/mg protein, *p* > 0.05). However, protein oxidation was very rapid during refrigeration (4 °C), and the highest values of carbonyl content (5.69–8.40 mmol/mg protein) were reached in all groups of surimi at 5 d. Among them, the carbonyl content of W0 increased rapidly, and the carbonyl content of W1, W2, and W3 increased more slowly. Li et al. [4] showed that the unwashed surimi had rapid protein oxidation during storage because of the high iron hemoglobin that was contained in the unwashed minced, whereas the washed surimi had more stable protein oxidation during storage. Some of the fat, myoglobin, pro-oxidant enzymes, and reducing sugars, which induce protein oxidation, were removed in the washing, which was able to inhibit protein carbonylation of surimi to some extent [5]. It is undeniable that lipid oxidation is critical as well as protein oxidation, which is closely related to the flavor and color of surimi samples. In addition, lipid oxidation and protein oxidation also influence each other. In the presence of carbonyl and reducing sugars, the Maillard reaction can also occur in the surimi samples during subsequent processing, resulting in color changes [42,43]. Therefore, the changes of protein oxidation, lipid oxidation, and gel quality during storage of different surimi samples still need further study.

The combination of free radicals with hydrogen ions that are ionized by sulfhydryl groups causes the formation of disulfide bonds between (or within) protein molecules, resulting in a decrease in the total sulfhydryl content, and the change in the total sulfhydryl content also allows the evaluation of protein oxidation [44]. Figure 4b shows that the total sulfhydryl content of all four surimi groups showed a decreasing trend during storage, indicating that some sulfhydryl groups were converted to form disulfide bonds and protein oxidation occurred. During storage, oxygen continuously combined with protein, which in turn promoted the oxidation of protein. The sulfhydryl content decreased faster at 0–2 d and more slowly thereafter, which is consistent with the study of Eymard, Baron, and Jacobsen [5]. The sulfhydryl content of surimi tended to decrease as the washing times increased and washing significantly reduced the sulfhydryl content compared to W0 (*p* < 0.05). The head and tail of myosin include several reactive sulfhydryl groups. When the conformation of myosin changes, its reactive sulfhydryl groups might be exposed. Washing promotes the unfolding of the protein spatial structure, allowing buried sulfhydryl groups to be exposed [7].

### 3.8. SDS-PAGE Profiles of Surimi

**Figure 5:**
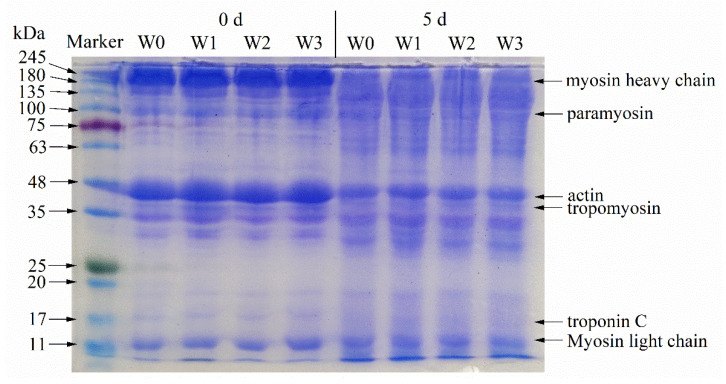
SDS-PAGE of surimi with different washing times during 4 °C refrigeration.

Myosin, actin, and actomyosin are the most important proteins in myofibrillar protein [6]. Figure 5 shows the SDS-PAGE of surimi with different washing times during 4 °C refrigeration (0 and 5 d). The lanes of the electropherogram correspond to Marker, W0 (0 d of storage), W1 (0 d of storage), W2 (0 d of storage), W3 (0 d of storage), W0 (5 d of storage), W1 (5 d of storage), W2 (5 d of storage), and W3 (5 d of storage), respectively. The results showed that large molecular proteins (MW > 200 kDa) accumulated at the top of the separated gel at 0 d due to hydrophobic interaction. Myosin is a salt-soluble protein and its content was not affected by washing with water, so there was no significant difference in the intensity of the MHC bands of surimi with different washing times. The protein bands appeared below 25 kDa in all the groups, indicating the presence of myosin in surimi at different washing times. However, some smaller fish protein fractions, such as troponin C, were removed during the washing process [21]. After 5 d, protein molecules were degraded, as evidenced by the blurring, weakening, expansion, and disappearance of high molecular weight protein bands and the appearance or concentration enhancement of low molecular weight bands [45]. With the extension of storage time, the cross-linking of proteins caused fading of the bands of MHC, MLC, and paramyosin. Li et al. [4] showed that oxidation of surimi protein was generated by disulfide bonds causing the cross-linking of proteins, and the degree of oxidation was related to the processing method and the time of storage. MHC and actin were the main proteins for the gelation of surimi. The concentration of this band in surimi decreased after storage, indicating that storage was not conducive to gelation of surimi.

## 4. Conclusions

Increasing the washing times improved the physicochemical characteristics and delayed the protein oxidation of silver carp surimi, and W2 and W3 were considered to be the most suitable washes for surimi gels with the most gel strength, WHC, and best microstructure, as well as less fat oxidation and protein oxidation, and also significantly reduced the amount of water that was used for rinsing. On the other hand, the surimi yield of W2 was higher than that of W3. In practice, two washing times can be used to produce silver carp surimi to improve the quality of surimi gel. However, the effects of different rinsing treatments on protein oxidation, lipid oxidation, and gel properties during frozen storage of surimi need further study. While less washing treatment can improve the yield of surimi, the addition of antifreeze and antioxidants is still required to improve the quality of frozen surimi.

## Figures and Tables

**Table 1:** Basic composition of surimi with different washing times.

	Moisture Content (%)	Protein Content (%)	Lipid Content (%)	Ash Content (%)
W0	73.14 ± 0.98 ^a^	24.71 ± 0.65 ^b^	1.52 ± 0.06 ^c^	0.53 ± 0.03 ^a^
W1	74.00 ± 0.91 ^ab^	24.19 ± 0.53 ^b^	1.24 ± 0.04 ^b^	0.53 ± 0.04 ^a^
W2	75.44 ± 0.32 ^bc^	22.87 ± 0.39 ^a^	1.19 ± 0.06 ^ab^	0.53 ± 0.02 ^a^
W3	76.30 ± 0.46 ^c^	22.21 ± 0.44 ^a^	1.10 ± 0.04 ^a^	0.52 ± 0.02 ^a^

Note: W0, W1, W2, and W3 represent the surimi with 0 washing times, 1 washing time, 2 washing times, and 3 washing times, respectively. Different letters in the same line indicate significant differences (*p* < 0.05).

**Table 2:** Fatty acid composition of surimi with different washing times.

Fatty Acid Composition (%)	W0	W1	W2	W3
C12:0	0.16 ± 0.00 ^c^	0.71 ± 0.01 ^d^	0.11 ± 0.00 ^b^	nd ^a^
C13:0	20.10 ± 0.09 ^c^	20.74 ± 0.21 ^d^	18.42 ± 0.23 ^b^	15.26 ± 0.17 ^a^
C14:0	3.08 ± 0.01 ^b^	2.99 ± 0.03 ^a^	7.76 ± 0.00 ^c^	9.33 ± 0.01 ^d^
C16:0	14.71 ± 0.00 ^b^	14.50 ± 0.02 ^a^	14.52 ± 0.09 ^a^	14.66 ± 0.10 ^ab^
C17:0	1.07 ± 0.00 ^b^	1.22 ± 0.00 ^c^	nd ^a^	nd ^a^
C18:0	2.22 ± 0.00 ^b^	3.71 ± 0.01 ^d^	2.78 ± 0.00 ^c^	2.14 ± 0.05 ^a^
C20:0	4.42 ± 0.00 ^b^	nd ^a^	nd ^a^	nd ^a^
C22:0	0.24 ± 0.00 ^b^	0.29 ± 0.00 ^c^	nd ^a^	nd ^a^
**∑Saturated**	**46.00 ± 0.09 ^d^**	**44.15 ± 0.16 ^c^**	**43.60 ± 0.13 ^b^**	**41.40 ± 0.12 ^a^**
C14:1	1.34 ± 0.00 ^d^	0.87 ± 0.00 ^c^	0.71 ± 0.01 ^b^	0.51 ± 0.01 ^a^
C16:1	9.55 ± 0.00 ^b^	9.42 ± 0.01 ^b^	9.02 ± 0.06 ^a^	11.55 ± 0.17 ^c^
C18:1	17.56 ± 0.21 ^a^	18.84 ± 0.15 ^b^	19.66 ± 0.20 ^c^	19.55 ± 0.29 ^c^
C20:1	2.54 ± 0.01 ^a^	3.07 ± 0.02 ^d^	2.94 ± 0.04 ^c^	2.72 ± 0.04 ^b^
C24:1	1.62 ± 0.00 ^d^	1.17 ± 0.00 ^a^	1.29 ± 0.00 ^c^	1.20 ± 0.01 ^b^
**∑Monounsaturated**	**32.62 ± 0.21 ^a^**	**33.37 ± 0.18 ^b^**	**33.62 ± 0.22 ^b^**	**35.53 ± 0.08 ^c^**
C18:2	2.61 ± 0.02 ^a^	3.22 ± 0.01 ^c^	3.22 ± 0.01 ^c^	2.98 ± 0.01 ^b^
C18:3n6	4.22 ± 0.08 ^a^	4.83 ± 0.01 ^b^	4.74 ± 0.03 ^b^	4.73 ± 0.05 ^b^
C20:2	0.35 ± 0.00 ^b^	0.43 ± 0.00 ^c^	nd ^a^	nd ^a^
C20:3n3	1.62 ± 0.00 ^a^	1.82 ± 0.01 ^b^	1.94 ± 0.01 ^c^	2.14 ± 0.02 ^d^
C20:4	5.25 ± 0.00 ^a^	5.60 ± 0.01 ^b^	6.26 ± 0.10 ^c^	6.86 ± 0.05 ^d^
C20:5 (EPA)	4.23 ± 0.02 ^d^	4.07 ± 0.00 ^c^	3.85 ± 0.13 ^b^	3.52 ± 0.06 ^a^
C22:6 (DHA)	3.09 ± 0.05 ^c^	2.53 ± 0.06 ^a^	2.77 ± 0.07 ^b^	2.85 ± 0.01 ^b^
**∑polyunsaturated**	**21.23 ± 0.12 ^a^**	**22.49 ± 0.09 ^b^**	**22.78 ± 0.36 ^bc^**	**23.08 ± 0.20 ^c^**

Note: Nd indicates that the substance that was tested was not detected. Different letters in the same line indicate significant differences (*p* < 0.05).

**Table 3:** Physicochemical characteristics of surimi with different washing times.

	W0	W1	W2	W3
Appearance	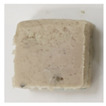	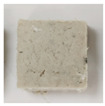	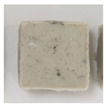	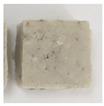
Yield (%)	100.00 ± 0.00 ^d^	88.58 ± 1.26 ^c^	82.65 ± 0.89 ^b^	76.73 ± 0.96 ^a^
Color (a*)	1.44 ± 0.44 ^a^	0.29 ± 0.49 ^b^	−1.31 ± 0.37 ^c^	−1.08 ± 0.39 ^c^
Whiteness	65.23 ± 1.03 ^a^	71.97 ± 1.10 ^b^	72.01 ± 0.89 ^b^	71.96 ± 0.80 ^b^
pH	6.32 ± 0.02 ^a^	6.35 ± 0.01 ^a^	6.66 ± 0.02 ^b^	6.83 ± 0.02 ^c^
TVB-N (mg N/100 g)	6.57 ± 0.24^c^	5.86 ± 0.46 ^bc^	5.27 ± 0.39 ^ab^	4.62 ± 0.35 ^a^
TBARS (mg/kg)	0.18 ± 0.00 ^d^	0.13 ± 0.00 ^c^	0.12 ± 0.00 ^b^	0.09 ± 0.00 ^a^
Breaking force (g)	369.67 ± 31.48 ^a^	403.29 ± 25.94 ^a^	419.33 ± 19.94 ^a^	413.67 ± 35.20 ^a^
Deformation (cm)	0.86 ± 0.04 ^a^	0.92 ± 0.03 ^ab^	0.97 ± 0.04 ^bc^	1.00 ± 0.01 ^c^
Gel strength (g·cm)	320.20 ± 38.60 ^a^	369.89 ± 32.94 ^b^	408.51 ± 36.03 ^c^	415.18 ± 35.68 ^c^
WHC (%)	88.46 ± 1.49 ^a^	90.90 ± 1.39 ^b^	92.53 ± 2.02 ^b^	92.87 ± 1.75 ^b^

Note: Different letters in the same line indicate significant differences (*p* < 0.05).

**Table 4:** Secondary structure of surimi with different washing times.

Secondary Structure	W0	W1	W2	W3
*α*-helical (%)	30.87 ± 0.24 ^a^	31.30 ± 0.34 ^a^	31.51 ± 0.26 ^a^	31.91 ± 0.29 ^a^
*β*-sheet (%)	44.06 ± 0.37 ^b^	42.88 ± 0.31 ^a^	41.85 ± 0.28 ^a^	42.62 ± 0.29 ^a^
*β*-turn (%)	12.18 ± 0.17 ^a^	12.62 ± 0.19 ^a^	13.52 ± 0.14 ^a^	12.69 ± 0.18 ^a^
Random coil (%)	12.88 ± 0.20 ^a^	13.20 ± 0.22 ^a^	13.12 ± 0.16 ^a^	12.78 ± 0.16 ^a^

Note: Different letters in the same line indicate significant differences (*p* < 0.05).

**Table 5:** Changes of T2 relaxation times of surimi gel with different washing times.

	W0	W1	W2	W3
*T*_2b_ (ms)	0.19 ± 0.00 ^a^	0.21 ± 0.01 ^b^	0.25 ± 0.01 ^c^	0.40 ± 0.01 ^d^
*T*_21_ (ms)	60.80 ± 0.00 ^a^	60.80 ± 0.00 ^a^	60.80 ± 0.00 ^a^	60.80 ± 0.00 ^a^
*T*_22_ (ms)	587.28 ± 0.00 ^a^	587.28 ± 0.00 ^a^	587.28 ± 0.00 ^a^	587.28 ± 0.00 ^a^
Peak areas *T*_2b_ (%)	2.78 ± 0.06 ^a^	3.66 ± 0.03 ^b^	3.74 ± 0.01 ^b^	3.86 ± 0.01 ^c^
Peak areas *T*_21_ (%)	93.18 ± 0.13 ^b^	90.85 ± 0.15 ^a^	90.95 ± 0.21 ^a^	90.56 ± 0.17 ^a^
Peak areas *T*_22_ (%)	4.05 ± 0.00 ^a^	5.49 ± 0.007 ^c^	5.31 ± 0.01 ^b^	5.57 ± 0.00 ^d^

Note: Different letters in the same line indicate significant differences (*p* < 0.05). *T*_2b_: relaxation time of bound water; *T*_21_: relaxation time of immobilized water; *T*_22_: relaxation time of free water. Peak areas *T*_2b_: percentage of *T*_2b_; Peak areas *T*_21_: percentage of *T*_21_; Peak areas *T*_22_: percentage of *T*_22_.

## Data Availability

Data is contained within the article or supplementary material.

## Supplementary Materials

The following supporting information can be downloaded at: https://www.mdpi.com/article/10.3390/foods11162397/s1, Figure S1: Gaussian curve fitting of amide Ι of surimi gel with different washing times.
