# Supplementary figures and images for: Effect of Washing Times on the Quality Characteristics and Protein Oxidation of Silver Carp Surimi
Source: Foods. 2022 Aug 10;11(16):2397. doi: 10.3390/foods11162397 (PMC9407351; doi:10.3390/foods11162397)

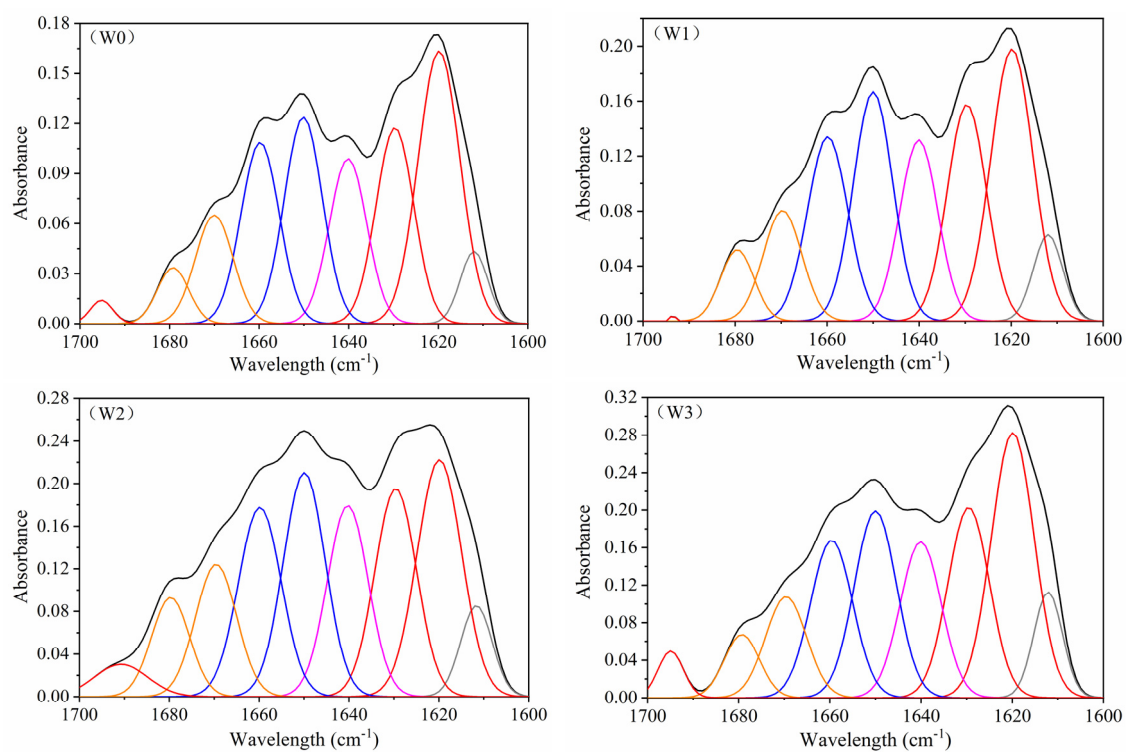

**Figure S1.** Gaussian curve fitting of amide I of surimi gel with different washing times.

Supplement: Supplementary file 1 [file foods-11-02397-s001.zip › foods-1823251-supplementary.pdf]
